# *In vitro* evaluation for estrogenic mechanisms of the disinfectant benzalkonium chloride as an emerging contaminant

**DOI:** 10.1590/1414-431X2023e12784

**Published:** 2023-07-21

**Authors:** Songyi Wei, Xianmin Hu, Xinyi Hu, Yisheng Wan, Guangquan Fan, Jun Wang

**Affiliations:** 1 Hubei Province Key Laboratory of Occupational Hazard Identification and Control, Wuhan University of Science and Technology, Wuhan, China

**Keywords:** Benzalkonium chloride, Disinfectant emerging contaminant, Estrogenic mechanisms, Xenoestrogen, Human adrenal H295R cell line, Estrogen receptor positive MCF-7 cell line

## Abstract

This study aimed to evaluate *in vitro* the possible mechanisms underlying the estrogenic potential of benzalkonium chloride (BAC) as a disinfectant emerging contaminant. Effects of BAC at the environmentally-relevant concentrations on estrogen synthesis and estrogen receptor (ER) signaling were assessed using the H295R steroidogenesis assay and the MCF-7 proliferation assay, respectively. Results showed that exposure to BAC at concentrations of 1.0-1.5 mg/L for 48 h significantly increased estradiol production of H295R cells in a concentration-dependent manner. Transcription of steroidogenic genes 3β‐HSD2, 17β‐HSD1, 17β‐HSD4, and CYP19A were significantly enhanced by BAC. In ER-positive MCF-7 cells, exposure to 0.5-1.5 mg/L BAC for 48 h significantly promoted cell proliferation and increased the expressions of ERα and G-protein coupled estrogen receptor 1. Flow cytometry analysis showed that 0.5-1.5 mg/L BAC significantly decreased the percentage of cells in G_0_/G_1_ phase, increased the percentage in S phase, and BAC at concentrations of 1.0 and 1.5 mg/L increased the G_2_/M phase cells. Findings of the study suggested that BAC at environmentally-relevant concentrations might act as a xenoestrogen through its inhibitory effect on steroidogenesis and ER-mediated mechanism.

## Introduction

To fight the global pandemic caused by the coronavirus disease 2019 (COVID-19), a number of public health strategies have been employed to halt the infection cycle. Among them, disinfection and hand hygiene using disinfectant products as high-touch surface cleaners and hand sanitizers are accepted best practices (1,2). Accordingly, increasing evidence (2-5) has demonstrated that the COVID-19 outbreak has triggered a sharp rise in the global consumption and use of disinfectants, which are expected to remain elevated during the ongoing pandemic. It has been forecasted that, from 2020 to 2027, the global surface disinfectant market will grow at a compound annual growth rate of 9.1% (6). However, under such conditions, concerns regarding adverse effects associated with disinfectants as emerging contaminants in the environment have recently been raised (2,7-10). As a result of heavy and frequent application of disinfection products during the COVID-19 pandemic, disinfectants will continuously enter the environment through multiple routes, including grey water discharge after application of household disinfectants as hand sanitizers or surface cleaners, improper disposal for unwanted or expired disinfectant products, hospital and municipal discharges, etc. (2,7). Based on bioactive and biocidal properties of disinfectants, the heavier environmental loads of disinfectant residues might pose non-negligible risks to ecosystems and human health (8-10).

Benzalkonium chloride (BAC) is one of the most common quaternary ammonium compounds (QACs), and BAC-based products are called the “workhorses” of modern disinfection as a group of cationic surfactants with powerful and broad-spectrum antimicrobial activity (7,11). Attention has been drawn to the environmental risks posed by BAC residues resulting from their continuous input and accumulation in the natural environment. In particular, recent studies highlighted the potential environmentally relevant estrogenic-disrupting potential of BAC. Proteomic analysis of whole-body responses in medaka (*Oryzias latipes*) exposed to BAC at concentrations of 0.05-0.2 mg/L for 21 days found that the expression of fatty acid-binding protein 3 (12), a biomarker of deleterious effects of endocrine disruptors in teleost testes and a potential target gene of estrogen-related receptor α (ERα), was significantly upregulated (13). Kim et al. (14) demonstrated that exposure to 3.0 μg/L BAC in the early-life stage of Japanese medaka (*O. latipes*) significantly upregulated the whole body gene transcription of *vitellogenin (vtg)* 1 and 2, biomarkers for estrogenic activity, by 5.8- and 11.3-fold, respectively, suggesting the endocrine disrupting potential of BAC residues in fish.

The most common mechanisms of estrogenic-disrupting chemicals include regulation of steroidogenesis and estrogen receptor (ER)-mediated responses (15). In order to verify the environmentally relevant estrogenic potential of BAC-based disinfectant as an emerging contaminant, the present study assessed the *in vitro* effects of BAC on estrogen synthesis and ER signaling using the H295R steroidogenesis assay and the MCF-7 proliferation assay, respectively.

## Material and Methods

### Chemicals

A mixture of BAC-C12 (73.3%), BAC-C14 (26.3%), and BAC-C16 (0.4%) (CAS No. 8001-54-5) was obtained from China National Institute for Food and Drug Control (China). Stock solutions of BAC were dissolved in dimethyl sulfoxide (DMSO) at a final concentration of 0.1% (v/v).

### H295R steroidogenesis assay

The human adrenal H295R cell line from ATCC (USA) was grown in DMEM medium (Sangon Biotech, China) for 5-8 passages. Then, the cells were seeded onto 96‐well plates at a density of 2×10^4^ cells/well to conduct the preliminary cell viability tests using MTT assay as previously described (16). The maximum exposure concentration of BAC without significant cytotoxicity was determined to be 1.5 mg/L.

H295R cells were seeded onto 24‐well plates at a density of 3×10^5^ cells/well for 17β-estradiol (E_2_) level assay or onto six‐well plates at a density of 1×10^6^ cells/well for gene expression assay. After incubation for 24 h, cells were exposed to BAC at concentrations of 0, 0.5, 1.0, and 1.5 mg/L for 48 h in triplicate (n=3).

After BAC exposure, for quantification of E_2_ levels, culture media were collected and E_2_ levels were measured using commercial E_2_ ELISA kits (Elabscience Biotechnology, China) following the manufacturer's instructions. E_2_ production is reported as fold above the vehicle control.

For the expression assay of genes involved in estrogen synthesis, trizol reagent (Invitrogen, USA) was added to the cell pellets to isolate total RNA. After cDNA synthesis, real-time PCR was performed to measure the expression of steroidogenic genes for estrogen synthesis including 3β‐hydroxysteroid dehydrogenase (HSD) 2, 17β‐HSD1, 17β‐HSD4, cytochrome P450 (CYP) 11A, CYP17, CYP19A, 3‐hydroxy‐3‐methyl‐glutaryl‐CoA reductase (HMGR), and steroidogenic acute regulatory protein (StAR). The details of primer sets and thermal cycle condition were as previously described (16-18). The relative amount of target mRNA was normalized to the amount of β-actin as internal control in the same sample.

### MCF-7 proliferation assay

Estrogen-responsive human breast cancer MCF‐7 cells purchased from the China Center for Type Culture Collection were starved in steroid‐free DMEM medium for 7 days to deplete intracellular estrogen storage. Then, the cells were allowed to attach onto 96‐well plates at a density of 1×10^4^ cells/well for the CCK-8 test to determine cell proliferation (n=5) or onto 6‐well plates at a density of 1×10^6^ cells/well for cell cycle analysis and western blot (n=3). After 24 h synchronization in steroid-free and serum-free medium, MCF‐7 cells were treated with BAC at concentrations of 0, 0.5, 1.0, and 1.5 mg/L for 48 h. Then, the CCK-8 reagents (Beyotime, China) were added and cells were incubated for an additional 2 h at 37°C. Absorbance at a wavelength of 450 nm was detected using a Multiscan MK3 plate reader (Thermo Scientific, USA). The cell proliferative effect is reported as a fold of the control.

In addition, cell suspensions were prepared and stained with propidium iodide (Sigma-Aldrich, USA) at 4°C for 30 min. Cell cycle analysis was performed using a Flow Cytometer and CellQuest Pro software version 5.1 (both from BD Biosciences, USA).

Protein expression levels of ERα and G-protein coupled estrogen receptor (GPER)1 in MCF‐7 cells were measured by western blot analysis. Total protein was extracted from cells using RIPA protein lysate (Shanghai Beyotime Biotechnology Co., Ltd., China). After protein assay, equal quantities of cell protein (50 µg/lane) were separated by SDS-PAGE on a 12% gel and then transferred by electroblotting onto a nitrocellulose membrane. The membrane was incubated with the primary antibodies of anti-ERα, anti-GPER1 (ABclonal, China), and β-actin (Santa Cruz, USA). Protein expression levels of ERα and GPER1 were corrected with the amount of β-actin in the same sample based on band intensities.

### Statistical analysis

Results are reported as the means±SD and were analyzed by one-way ANOVA with a *post hoc* Tukey test using SPSS 24.0 software (USA). A P value less than 0.05 was considered statistically significant.

## Results

### Effects of BAC exposure on estrogen production and mRNA expression of steroidogenic genes for estrogen synthesis in H295R cells

**Figure 1:**
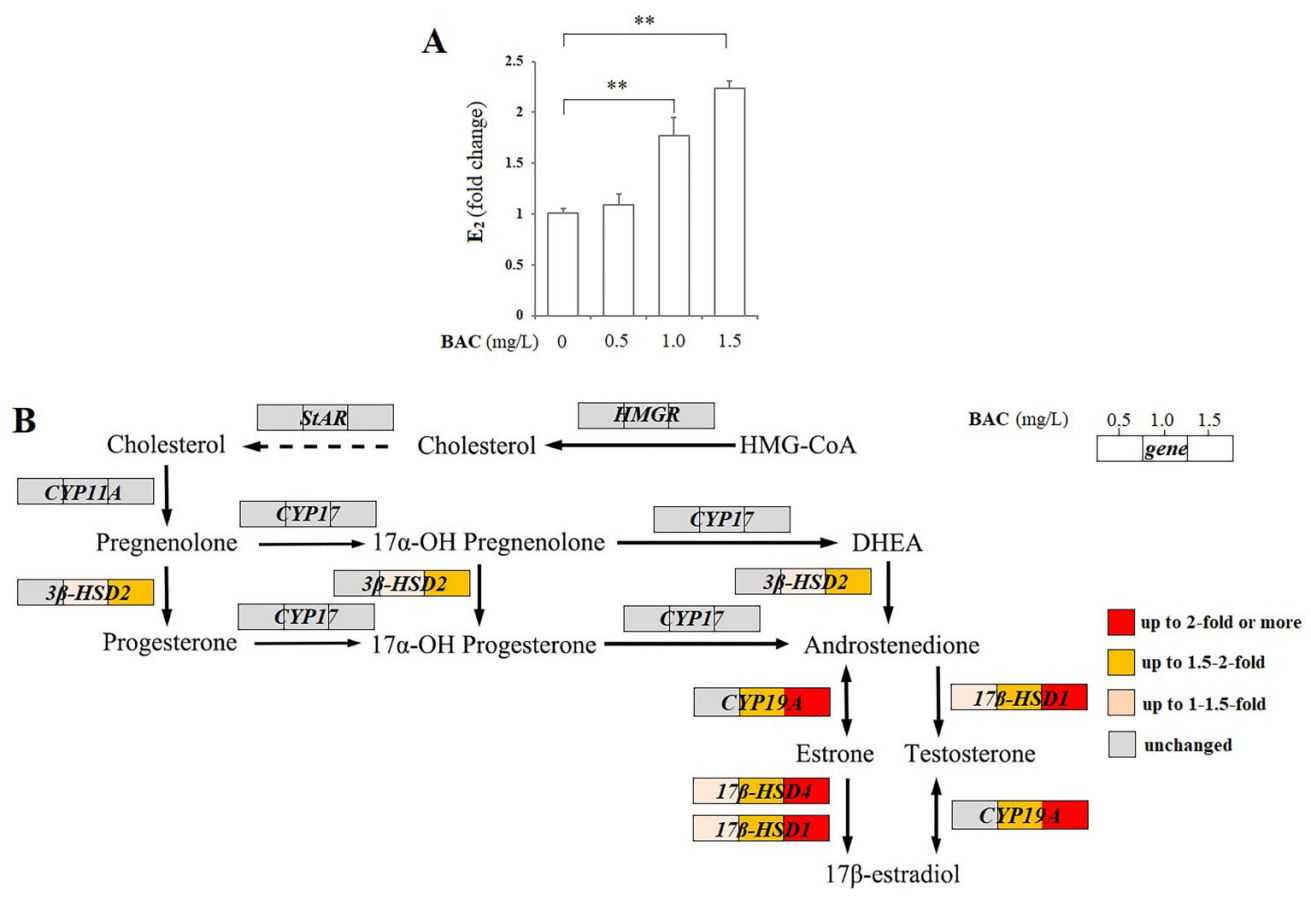
17β-estradiol (E_2_) production (**A**) and expression of steroidogenic genes for estrogen synthesis (**B**) in H295R cells exposed to benzalkonium chloride (BAC) (0.5, 1.0, 1.5 mg/L) (n=3). Data are reported as means±SD. **P<0.01 compared to the vehicle controls (ANOVA). The colors indicate different fold thresholds.

As shown in Figure 1A, exposure to BAC at concentrations of 1.0-1.5 mg/L significantly increased E_2_ levels in culture supernatants of H295R cells (P<0.01). Compared with the control cells, estrogen biosynthesis was significantly enhanced by 1.73‐fold and 2.22‐fold in 1.0 and 1.5 mg/L BAC exposure groups, respectively. This concentration‐dependent increase of estrogen production induced by BAC exposure suggested the estrogenic activity of BAC at environmentally relevant concentrations.

To further investigate the possible mechanisms involved in BAC-induced estrogen steroidogenesis, we analyzed the mRNA expression of steroidogenic genes involved in estrogen synthesis, including 3β‐HSD2, 17β‐HSD1, 17β‐HSD4, CYP11A, CYP17, CYP19A, HMGR, and StAR in H295R cells exposed to BAC. As shown in Figure 1B and Table 1, exposure to BAC only caused significant upregulations in mRNA expression levels of three HSDs and CYP19A in a concentration-dependent manner (P<0.05; P<0.01, respectively). In the 1.5 mg/L BAC exposure group, the transcriptions of steroidogenic genes 3β‐HSD2, 17β‐HSD1, 17β‐HSD4, and CYP19A were significantly enhanced by 1.8-fold, 2.3-fold, 2.1-fold, and 2.0-fold relative to the controls, respectively.

**Table 1:** Transcriptional response profiles of steroidogenic genes for estrogen synthesis in H295R cells exposed to benzalkonium chloride (BAC).

Gene	BAC exposure			
	0 mg/L	0.5 mg/L	1.0 mg/L	1.5 mg/L
*HMGR*	1.01±0.02	0.96±0.23	1.03±0.22	1.24±0.36
*StAR*	1.02±0.03	1.12±0.31	0.91±0.34	1.10±0.27
*CYP11A*	1.00±0.02	1.28±0.24	1.27±0.30	1.41± 0.36
*CYP17*	0.98±0.15	1.15±0.25	1.18±0.27	1.22±0.32
*CYP19A*	1.00±0.06	1.42±0.52	1.72±0.34*	2.01±0.37**
*3β-HSD2*	1.01±0.05	1.28±0.21	1.42±0.29*	1.78±0.31**
*17β-HSD1*	1.02±0.05	1.37±0.16*	1.50±0.18**	2.32±0.13**
*17β-HSD4*	1.02±0.08	1.33±0.16*	1.70±0.27**	2.14±0.19**

The mRNA expression is reported as the means±SD fold change compared to the vehicle controls (n=3). *P<0.05, **P<0.01 compared to the vehicle controls (0 mg/L) (ANOVA).

### Effects of BAC exposure on cell proliferation, cell cycle progression, and expression of ERs in MCF-7 cells

**Figure 2:**
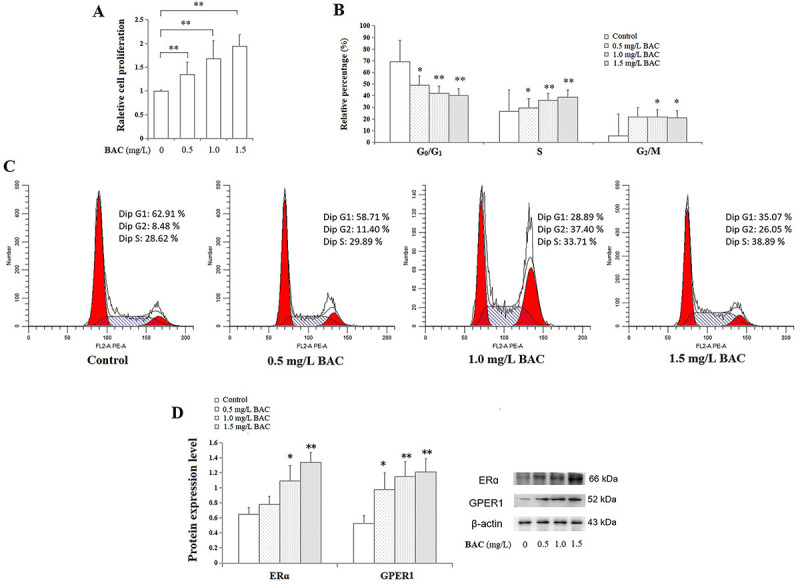
Cell proliferation (**A**), cell cycle (**B**), representative flow cytometry histograms showing cell cycle distributions (**C**), and estrogen receptor (ER)α and G-protein coupled estrogen receptor 1 (GPER1) expressions (**D**) of MCF-7 cells exposed to benzalkonium chloride (BAC) (0.5, 1.0, 1.5 mg/L). Data are reported as means±SD. *P<0.05, **P<0.01 compared to the vehicle controls (ANOVA).

As shown in Figure 2A, compared to the controls, exposure of MCF-7 cells to concentrations of BAC ranging from 0.5 to 1.5 mg/L for 48 h significantly increased the cell proliferative rate by 1.35-1.95 fold in a concentration-dependent manner (P<0.01). Moreover, cell cycle analysis showed that exposure to 0.5-1.5 mg/L BAC significantly decreased the cell percentage in G_0_/G_1_ phase and enhanced the percentage in S phase (P<0.05; P<0.01, respectively), and BAC at concentrations of 1.0 and 1.5 mg/L increased the G_2_/M phase cells (P<0.05) (Figure 2B and C), suggesting BAC might have a capacity to drive MCF-7 cells into S phase. The S phase cell percentage in the 1.5 mg/L BAC exposure group increased by approximately 1.5-fold compared with the controls. As shown in Figure 2D, the protein expression levels of ERα and GPER1 were significantly enhanced by 0.5-1.5 mg/L BAC (P<0.05; P<0.01, respectively). In the MCF-7 cells exposed to 1.5 mg/L BAC, the expressions of ERα and GPER1 were 2.1-fold and 2.3-fold of the controls, respectively.

## Discussion

Before the pandemic, more than one million pounds per year of BAC had already been manufactured or imported into the United States, thus the Environmental Protection Agency (EPA) included BAC in its High Production Volume list (7,11). Importantly, growing evidence (19,20) has shown a fast and potent antiviral effect of BAC through the disruption of the lipid bilayer envelope of coronaviruses including SARS-CoV-2, the agent of COVID-19. So far, the EPA of the United States, Health Canada (20), and the new Chinese guidelines for use of disinfectants include BAC products in the national official lists of disinfectants recommended for application against SARS-CoV-2. As an effective alternative to alcohol-based disinfectant products, BAC is non-toxic, non-flammable, and less irritating to the skin, thus widely utilized in alcohol-free hand sanitizers, antibacterial soaps, cleaning wipes, laundry detergents, surface disinfectants, hospital sanitation kits, etc. (19). Because of its wide use, the environmental impact of BAC as an emerging contaminant cannot be disregarded (11) and will continue to rise due to the pandemic. Even before the COVID-19 outbreak, the levels of BAC in surface water samples have been reported to range from 0.04 to 342 μg/L (14,21,22). In hospital effluents, relatively higher levels of BAC ranging from 0.05 to 6.03 mg/L and from 1.65 to 3.93 mg/L were found in Europe (23) and Austria (24), respectively. Moreover, because of its strong absorbability, considerable stability to hydrolysis, photodegradation, and biodegradation (6,25), BAC has been frequently reported to occur in solid environmental matrices at quite high levels. In samples of municipal sewage sludge from China, the total concentration of BAC was in the range of 0.09-191 μg/g dry weight (26). In Swedish sewage sludge, the highest average value for BAC residual levels was 35 mg/g dry weight (25).

However, especially since BAC was suspected as the cause of a social disaster related to household humidifier disinfectants in Korea in 2011 (27), toxicological studies on BAC have attracted considerable attention. So far, there is available data on corneal and nasal toxicity, neurotoxicity, and genotoxicity of BAC (11). The reproductive toxicity and endocrine disrupting potential of QACs have been demonstrated in mice orally exposed to the mixture of BAC and the QAC didecyl dimethyl ammonium chloride (28,29). More importantly, evidence in fish has shown that BAC at environmentally relevant concentrations might upregulate the expression of some estrogen-related molecules, such as fatty acid-binding protein 3 and vitellogenin (12,14). In particular, Kim et al. (14) confirmed that, in line with the up-regulation of vitellogenin gene in early stage zebrafish, a significantly increased E_2_ level was found in H295R cells exposed to BAC at a single concentration of 10 μM (3.39 mg/L). However, in the present study, we found a significant cytotoxicity of BAC above the concentration of 1.5 mg/L. The reason for this difference might be due to different mixtures of BAC products used in the two studies. A mixture of BAC-C12 (70%) and BAC-C14 (30%) purchased from Sigma Aldrich (USA) was used in the study conducted by Kim et al. (14), while our study used a mixture of BAC-C12 (73.3%), BAC-C14 (26.3%), and BAC-C16 (0.4%) from China National Institute for Food and Drug Control. Nevertheless, consistent with the finding from Kim et al. (14), the present study showed a concentration-dependent induction of estrogen synthesis in H295R cells by BAC exposure at concentrations of 0.5-1.5 mg/L, which were within the range of values obtained from measured environmental concentrations of BAC reported in hospital effluents (23,24), thus confirming the estrogenic potential of BAC as a contaminant.

To explore the mechanism underlying the estrogenic potential of BAC, we further detected the mRNA expression of steroidogenic enzymes involved in estrogen biosynthesis in H295R cells. As an immortal adrenergic carcinoma cell line which maintains the ability to express all the enzymes and their genes of the steroidogenic pathways, the H295R cell has been well-accepted as a standard *in vitro* model to explore endocrine disrupting potency and mechanisms of toxicants and contaminants (18). Estrogen is synthesized from cholesterol *via* sequential reactions catalyzed by a series of steroidogenic enzymes. In this study, we measured the effect of BAC exposure on the eight steroidogenic genes involved in estrogen synthesis, including three genes encoding cytochrome P450 enzymes (CYP11A, CYP17, CYP19A), three genes encoding hydroxysteroid dehydrogenases (3β-HSD2, 17β‐HSD1, 17β‐HSD4), and two genes for cholesterol biosynthesis and transport (HMGR, StAR). The results showed that BAC exposure promoted estrogen synthesis by significantly enhancing mRNA expression of three genes encoding hydroxysteroid dehydrogenases (3β‐HSD2, 17β‐HSD1, 17β‐HSD4) and CYP19A in a concentration-dependent manner, but had no statistically significant effect on genes encoding CYP11A and CYP17 and for cholesterol biosynthesis/transport. The 17β‐HSDs and CYP19A in H295R cells appeared to be sensitive to BAC exposure. As important downstream enzymes in steroidogenesis, 17β‐HSD1 and 17β‐HSD4 play crucial roles in controlling the last step in the formation of the essential active estrogens (18). As the rate-limiting enzyme and the major pathway of estradiol production, CYP19A is the terminal steroidogenic enzyme responsible for final conversion of androgens to estrogens, a process critical for sex differentiation and adult reproductive cycles (18).

On the other hand, it has been demonstrated that environmental endocrine-disrupting chemicals, for example, bisphenol A and bisphenol S, exert their hormone-like effects *via* an ER-mediated mechanism (30-32). The ER-positive MCF-7 cell line expressing ERα and GPER1 is sensitive to hormones and widely used in the *in vitro* assessment of estrogenic potential and its mechanisms of environmental xenoestrogens (30-34). In this study, the proliferative effect of BAC at concentrations of 0.5-1.5 mg/L and increased ERα and GPER1 expression were observed in MCF-7 cells, suggesting that BAC at environmentally-relevant concentrations might act as an ER agonist to pose potential endocrine disrupting risks. Accordingly, BAC decreased the percentage of MCF-7 cells at G_0_/G_1_ phase and increased the percentage of cells at S phase, thus promoting cell cycle progression, which was in line with the findings that xenoestrogens, such as bisphenol A, bisphenol S, and microplastic-derived plasticizers, accelerated the G_1_-S phase transition in MCF-7 cells (31,33,34). However, in the presence of E_2_, based on the recombinant human breast carcinoma VM7Luc4E2 cells containing a stably integrated ER-responsive firefly luciferase reporter plasmid, a previous study (35) showed a weak antiestrogenic effect of BAC by means of a luciferase-based assay. This discrepancy suggested that BAC appears to exert a bi-directional estrogenic regulation in the presence or absence of E_2_. Moreover, the IC_50_ value for BAC was determined to be 17.3 µM (about 6.5 mg/L) for its antiestrogenic activity in the study conducted by Datta et al. (35), but the concentration range of BAC in the present study was 0.5-1.5 mg/L. In addition, the differences in manufacturers and compositions of BAC products might also contribute to the discrepancy. In fact, the BAC exists as a mixture of alkylbenzyl dimethyl ammonium chlorides with different even-number alkyl chain lengths. It has been found that BAC with a different alkyl chain length showed different biological activity, such as the inhibitor effect on cholesterol biosynthesis (36). Therefore, further studies considering all the above aspects would help to clarify the estrogenic-disrupting potential of BAC as an environmental contaminant.

In conclusion, the data suggested that BAC at environmentally-relevant concentrations might act as a xenoestrogen through its inhibitory effect on steroidogenesis and ER-mediated mechanism.
